# Prevalence of Malocclusion in Permanent Dentition of Iranian Population: A Review Article

**Published:** 2018-02

**Authors:** Faezeh ESLAMIPOUR, Zohreh AFSHARI, Arash NAJIMI

**Affiliations:** 1. Dental Research Center, Dept. of Oral Health, School of Dentistry, Isfahan University of Medical Sciences, Isfahan, Iran; 2. Dental students’ Research Center, School of Dentistry, Isfahan University of Medical Sciences, Isfahan, Iran; 3. Dept. of Medical Education, Medical Education Research Center, Isfahan University of Medical Sciences, Isfahan, Iran

**Keywords:** Dental malocclusion, Prevalence, Angle classification, Iran

## Abstract

**Background:**

The aim of this study was systematic review and meta-analysis of prevalence in current and relevant literature about this developmental disorder to present the profile of malocclusion in Iran.

**Methods:**

This review study was carried out with systematically identified and critically assessed studies reporting malocclusion prevalence among Iranian population in permanent dentition. National and international databases were searched for articles about prevalence of malocclusion by Angle classification in different regions of Iran from 1994 to 2015. After applying inclusion and exclusion criteria, the quality of articles was checked by professional checklist. Data extraction and meta-analysis was performed. A random-effect model was employed. Publication bias was checked.

**Results:**

Of 2768 articles, 21 cases were included. The pooled prevalence of malocclusion was about 87% (95% CI: 78.3–92.2) in Iranian population; however, the prevalence of malocclusion across individual studies varied considerably (ranging from 23.7% to 99.7%). Prevalence of normal occlusion, class I, II and III malocclusion were reported as 13.3% (CI 95%: 7.8–21.7), 50.7% (CI 95%: 42.9–58.4), 21% (CI 95%: 17.5–25.1), 5.5% (CI 95%: 3–10); respectively. Maximum prevalence of malocclusion was in the East of Iran.

**Conclusion:**

The results showed a high prevalence of malocclusion in Iranian population. The baseline information could be appropriately utilized for the future planning to meet the orthodontic treatment need among the Iranian population.

## Introduction

Malocclusion is the most common developmental disorder that has significant negative impact on both children’s and their families’ quality of life (1–3). It is leading to psychosocial distress, speech and chewing problems (4), raising the possibility of injury in accidents (5, 6), periodontal problems (7), temporomandibular joint disease (8), bruxism (9), and headache (10). The prevalence and severity of malocclusion have increased over the last centuries. Therefore, needs for orthodontic treatment have been increased (11).

Determining the prevalence of malocclusion in a specific population group can help in developing health policies to prevent and treat (12). For evaluation the prevalence of malocclusion, we need to universal acceptable classification. Angle established a classification of occlusion based on the molar relationship still used today (13).

A literature review showed different prevalence reports (23.7% to 99.7%) for dental malocclusion in various areas of Iran. About 78% of 1063 adolescence in Karaj had malocclusion (14). In 6151 children, 6 to 17 yr-old in Isfahan only 15% had normal occlusion and 85.2% were categorized into different types of malocclusions (15). In Shiraz, among 1338 people between 14 and 18 yr old, 23.7% had different types of malocclusions (16). In Tehran, 99.7% of 12 to 15 yr-old children had malocclusion (17).

The present study was aimed to investigate the pooled prevalence of malocclusion using all available national data.

## Methods

This meta-analysis study with regard prevalence of malocclusion was performed from 1994 to 2015 in Iran.

**Search strategy:** Electronic search was performed using international database including PubMed, Scopus, Google Scholar and national database including Iranian Scientific Information Database (SID), indexing article published in Iranian biomedical journals (Iran Medex), Iranian Research Institute of Information and Documentation (Iran Doc), Magiran and National Library and Archives from 1994 to 2015 in Iran. All thesis abstracts and original articles published up to Nov 2015 were put to work on. The search for articles was performed by two independent researchers using following search words: “malocclusion, Angle classification, class I malocclusion, class II malocclusion, class III malocclusion, prevalence, permanent dentition, dental malocclusion, cross-sectional studies and Iran”. The Persian keywords were equivalent to their English word, and all probable combinations were considered.

Moreover, manual search of reference lists of previously selected studies was carefully performed to gather additional scientific reports, also submitted to full-text examination. A gray literature search was also performed using google Scholar, Iran Doc, National Library, and Archives of Islamic Republic of Iran, Searching for available thesis, dissertations, and unpublished articles.

### Selection criteria

Two investigators in a two-step approach performed the selection process independently. After excluding duplicated articles, at first step, title and abstracts of all matched articles were reviewed for exclusion of unrelated articles. As inclusion criteria, descriptive studies that assessed prevalence of malocclusion in permanent dentition using Angle classification in Iranian population aged 11 to 35 yr were selected for review. Studies on syndromic or specific patients; assessment of other occlusal problems like cross bites, open bite and crowding and Studies were conducted on patients referred for orthodontic treatment to health centers were excluded. Disagreements were resolved by discussion.

### Quality Assessment

After selecting the articles, assessing risk of bias in studies was conducted by using a checklist based on STROBE protocol. This checklist contains 12 questions covered various aspects of the methodology such as sample size, study design, sampling method, population, data collection methods, and tools, examining samples method, statistical analysis and reporting findings based on objectives. Each items corresponded to “yes=1” or “no=0”. Therefore, each study was allocated a score of 0 to 12. If a study achieved less than 8 points it was omitted from meta-analysis (18).

### Data Extraction

The evidence from the selected studies was recorded into an evidence table that included the name of the first author, year of publication, the region where the study conducted, the total number of samples, the number of samples by sex if it had been reported, the prevalence of normal occlusion and malocclusions and various subdivisions if it had been reported. The studies were categorized into five geographic regions in Iran. Region 1: Isfahan and Yazd cities were used as the sample of the center of Iran, region 2: Tehran, Karaj, Qazvin and Rezvanshahr cities as North, region 3: Shiraz and Ahvaz cities as South sample, region 4: Mashhad, Kerman, Neishabour and Zahedan cities as the East and region 5: Mehran, Khorram Abad, and Tabriz cities were the West sample.

### Statistical analysis

Comprehensive meta-analysis (V2.2, Biostat) was used to conduct the meta-analysis. The pooled prevalence of Malocclusion among children was calculated using a random-effect model in light of the observed heterogeneity (19). The statistical heterogeneity within studies was evaluated using a χ^2^-based Cochran’s Q statistic (20), and was further quantified using *I^2^* statistics (*I^2^* =0%–25%, no heterogeneity; *I^2^* = 25%–50%, moderate heterogeneity; *I^2^* =50%–75%, large heterogeneity; and *I^2^* =75%–100%, extreme heterogeneity) (21). Sub-group analyses were conducted in accordance with the geographic areas (Center/ North /South/ East/ West), gender (male/female), types of malocclusion (normal occlusion/ class I malocclusion/ class II malocclusion/ class III malocclusion), and their subdivisions. Publication bias was assessed by funnel plot, Egger’s weighted regression, and Begg’s rank correlation methods (22).

After exclusion, a sensitivity analysis was performed to evaluate the effect of sample size on the pooled prevalence. If the outcome was significantly changed after one study was removed, then the study was excluded from the included studies due to selection bias, and a new analysis was conducted (19). *P*<0.05 was considered statistically significant for all analyses.

## Results

**Fig. 1:**
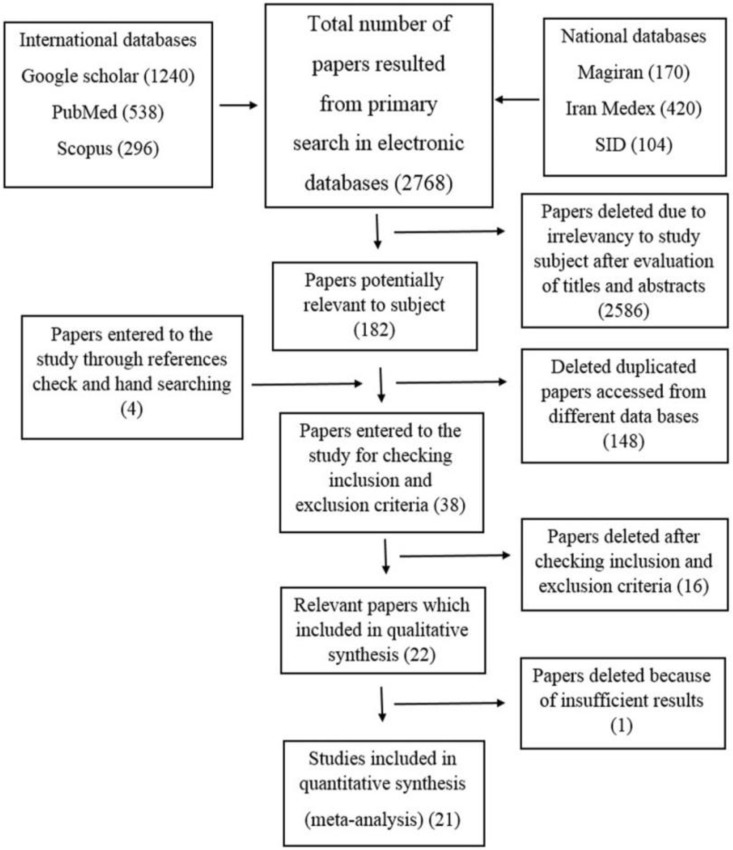
Searching flowchart

The original search in national and international databases identified 2768 publications which completed by 4 article of hand searching, of which 2734 were excluded after removal of duplications and review of the title and abstract. Application of the inclusion and exclusion criteria reduced these to 22 cross-sectional studies from 1994 to 2015 evaluated their full text and qualified for the review analysis (Fig. 1).

One study was excluded, mainly because the authors reported malocclusion in 6 to 17 aged population and were unable to provide exact data about prevalence of malocclusion in people with permanent dentition separately (18). Twenty-one eligible cross-sectional studies with a total number of 19498 participants met the inclusion criteria and were used for the meta-analysis (Table 1). Results of some studies were corrected using main data of research by making connection with correspondent author.

**Table 1:** Key characteristics of included studies in the meta-analysis of malocclusion prevalence in Iranian population

***Author (et al.)***	***Region***	***Total number***	***Male***	***Female***	***Normal***	***Class 1***	***Class2 div1***	***Class2 div2***	***Total class2***	***True class3***	***Pseudo class3***	***Total class3***	***Total malocclusion***
Ordubazari(17)	2	2519	1278	1241	0.3	62.1	21.7	15	36.7	NR	NR	0.9	99.7
Akhundi(14)	2	1063	1063	0	22.2	68.2	7.7	1.3	9	NR	NR	0.6	77.8
Ramezanzade(56)**	4	1000	500	500	4.7	55.1	12.3	5.7	18	7.3	0.3	7.6	95.3
Nuri(57)	2	1800	900	900	10.9	44.1	16	4.2	32.1	NR	NR	12.9	89.1
Akhundi(24)**	5	562	285	277	17.62	56.94	8.72	6.04	14.76	NR	NR	10.68	82.38
Ravanmehr(58)**	2	500	250	250	16	48	15.6	5.2	20.8	12	3.2	15.2	84
Hedayati(59)**	3	632	367	265	6.7	61.16	18.04	3.26	21.3	NR	NR	4.46	93.3
Ghodsi(60)**	1	960	480	480	10.73	44.06	NR	NR	29.06	NR	NR	16.15	89.27
Khanemasjedi(61)	3	744	744	0	2.82	62.91	26.34	2.82	29.16	NR	NR	5.11	97.18
Ramezanzade(62)**	4	469	254	215	13.6	54	16.4	6.8	23.2	5.8	3.4	9.2	86.4
Taheri(63)	2	300	150	150	8.7	NR	NR	NR	NR	NR	NR	NR	91.3
Shahri(23)**	4	630	315	315	7.9	76.9	12.7	1.4	14.1	0.6	0.5	1.1	92.1
Basafa(8)	4	425	308	117	18.8	43	12	7	19	NR	NR	19.2	81.2
Ahangar(64)	5	398	398	0	4	57	17.6	4.3	21.9	NR	NR	17.1	96
Mirzaei(65)	3	1338	621	717	38	31	16	12	28	NR	NR	3	62
Jafari(66)	2	1484	743	741	79*	NR	NR	14.5	NR	NR	6.5	NR	
Kuchmeshgi(67)	2	600	314	286	58.5	NR	NR	NR	NR	NR	NR	NR	41.5
Aghili(25)	1	1980	1980	0	51*	NR	NR	22	NR	NR	27	NR	
Borzabadi(68)**	1	502	249	253	22.9	41.8	24.1	3.4	27.5	NR	NR	7.8	77.1
Arabiun(16)**	3	1338	621	717	76.31	12.78	4.78	5.16	9.94	NR	NR	0.97	23.69
Fallahinezhad(69)**	5	254	124	130	30.7	49.64	7.4	5.9	13.3	5.5	0.7	6.2	69.3

NR: Not reported,

* Not used in meta-analysis of normal occlusion and class I malocclusion

** Included studies in the meta-analysis that reports prevalence of malocclusions by sex

### Meta-analysis Findings

**Fig. 2:**
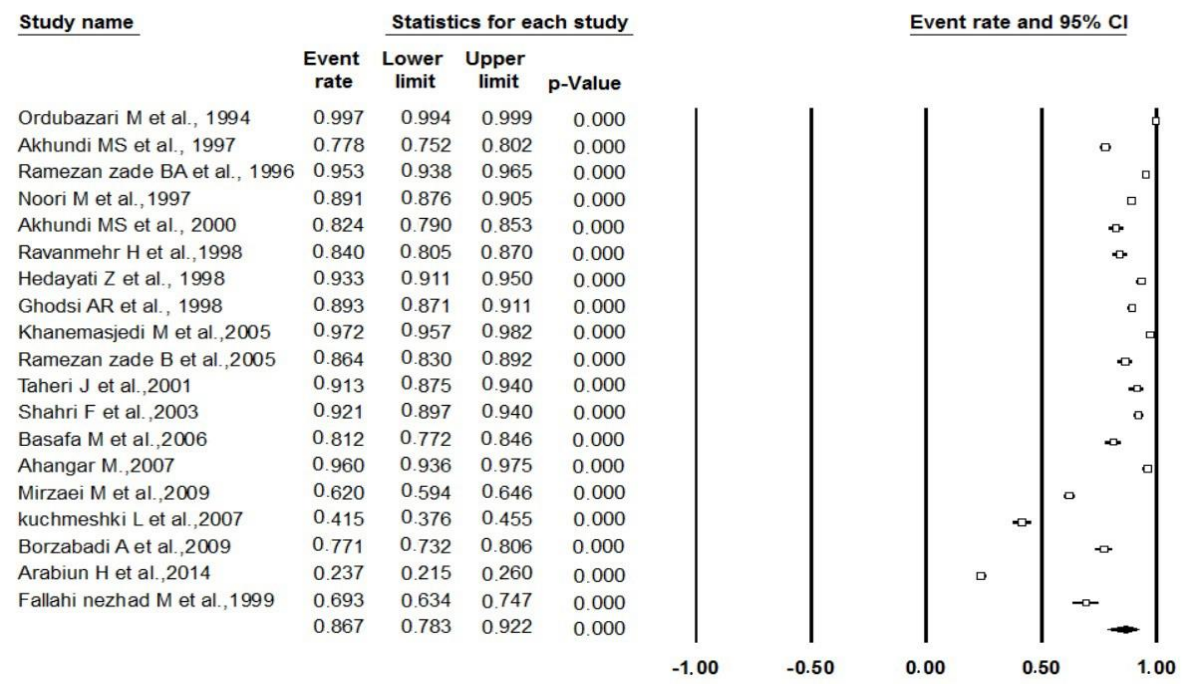
Forest plot of total malocclusion

Since the distribution of values in all of the meta-analyses exhibited significant heterogeneity, we used a random effect model for all meta-analyses. The prevalence of malocclusions in Iranian population was reported by all 21 included studies. Reported total malocclusion prevalence ranged from a low of 23.7% (7), to a high of 99.7% (17), and this range showed the extent of the variation in reported prevalence. The prevalence of class I malocclusion in articles varied from 12.7% (16), to 76.9% (23). The prevalence of class II malocclusion in articles varied from 9% (24), to 36.7% (17), and about class III malocclusion it was varied from 0.6% (24), to 27% (25). The pooled prevalence of malocclusion was 86.7% (95% CI: 78.3 – 92.2%) (Fig. 2). The most prevalent type of malocclusion in this study was class I malocclusion with 50.7% (95% CI: 42.9 – 58.4%). The pooled prevalence of class II malocclusion was 21% (95% CI: 17.5 – 25.1%). The meta-analysis showed that the total class III prevalence was 5.5 % (95% CI: 3 – 10%) (Table 2). Ten studies had been reported malocclusions based on gender. Meta-analysis based on these studies showed the prevalence of malocclusion in boys and girls were 83% (95%CI: 66.2–92.4%), 79.9% (95% CI: 62–90.6%); respectively. Although 16 articles have been reported the prevalence of subdivisions of class II and only 5 articles have been reported subdivisions of class III malocclusion, based on pooled data of these studies Class II div 1 (13.9%) was more prevalent than div II; and pseudo class III was the least prevalent malocclusions (1.2%) (Table 2).

**Table 2:** Pooled prevalence of malocclusion according to Angle classification, subdivisions of malocclusion and sexual distribution of malocclusions

***Variables***	***Total***			***Boys***			***Girls***		
	**Prevalence (95%CI)**	**Heterogeneity**		**Prevalence (95%CI)**	**Heterogeneity**		**Prevalence (95%CI)**	**Heterogeneity**	
		**Q value**	**I2 (%)**		**Q value**	**I2 (%)**		**Q value**	**I2 (%)**
Sample size (n)	19498			11945			7553		
CL I	50.7 (42.9, 58.4)	1340.32	98.80	50.3 (38.6, 61.9)	382.47	97.64	48.9 (36.1, 61.9)	452.96	98.013
Total CL II	21.0 (17.5, 25.1)	755.197	97.61	19.8 (15.9, 24.4)	86.25	89.56	18.0 (13.8, 23.0)	106.57	91.55
CL II,div1	13.9 (11.3, 17.0)	356.529	95.79	-	-	-	-	-	-
CL II,div2	4.8 (3.4, 6.8)	393.744	96.19	-	-	-	-	-	-
Total CL III	5.5 (3.0, 10.0)	1012.83	98.22	6.6 (4.3, 9.9)	94.69	90.49	6.3 (3.9, 10.1)	118.34	92.39
Pseudo CL III	1.2 (0. 5, 2.9)	26.904	85.13	-	-	-	-	-	-
True CL III	5.3 (3.1, 9.0)	42.962	90.68	-	-	-	-	-	-
Normal	13.3 (7.8, 21.7)	2740.188	99.34	17.0 (7.6, 33.8)	787.82	98.85	20.1 (9.4, 38.0)	825.56	98.91
Total malocclusion	86.7 (78.3, 92.2)	2740.188	99.34	83.0 (66.2, 92.4)	787.823	98.85	79.9 (62.0, 90.6)	825.56	98.91

The study geographic region was coded to show dispersion of types of malocclusions in North/ South/ East/ West and central area of Iran. The results showed the most and least prevalence of malocclusion in East (89.9%) and South (79.6 %); respectively.

Maximum prevalence of class I malocclusion (58%) was in Eastern area of Iran. The most prevalence of class II (26%) and class III (15.6%) malocclusion were in central region of Iran (Table 3). Significant publication bias was found in total malocclusion (*P*=0.007 by Begg's rank correlation test; *P*=0.001 by Egger's weighted regression analysis). In addition, class II and class III malocclusion were significantly heterogeneous (*P*<0.05 in both Egger and Begg's analysis). Trim and fill procedure was applied to correct for publication bias.

**Table 3:** Pooled prevalence of malocclusion according to geographic regions

***Variables***	***Region***	***Number of studies***	***Sample size (n)***	***Total***		
				**Prevalence (95%CI)**	**Heterogeneity**	
					**Q value**	**I2 (%)**
CL I	1	2	1462	43.3 (40.8, 45.8)	0.686	0
	2	4	5882	55.9 (44.2, 66.9)	214.761	98.60
	3	4	4052	39.3 (18.3, 65.1)	646.218	99.53
	4	4	2724	58.0 (44.2, 70.6)	130.972	97.70
	5	3	1214	55.0 (50.7, 59.2)	4.347	53.98
CL II	1	3	3442	26.0 (21.3, 31.2)	19.54	89.76
	2	5	7366	20.8 (12.7, 32.0)	410.874	99.02
	3	4	4052	20.9 (13.0, 31.7)	154.207	98.05
	4	4	2724	21.6 (14.3, 31.2)	77.603	96.13
	5	3	1214	16.5 (12.0, 22.3)	11.152	82.06
CL III	1	3	3442	15.6 (8.3, 27.5)	99.64	97.99
	2	5	7366	4.1 (1.7, 9.3)	243.918	98.36
	3	4	4052	3.0 (1.6, 5.3)	30.332	90.11
	4	4	2724	6.9 (3.2, 14.2)	80.583	96.27
	5	3	1214	10.9 (6.5, 17.7)	18.085	88.94
Normal occlusion	1	2	1462	15.9 (7.2, 31.4)	36.936	97.29
	2	6	6782	11.0 (4.4, 25.1)	692.832	99.27
	3	4	4052	20.4 (5.0, 55.6)	932.36	99.67
	4	4	2724	10.1 (5.4, 17.9)	72.63	95.87
	5	3	1214	14.0 (5.6, 30.9)	68.178	97.06
Total malocclusion	1	2	1462	84.1 (68.6, 92.8)	36.93	97.29
	2	6	6782	89.0 (74.9, 95.6)	692.83	99.27
	3	4	4052	79.6 (44.4, 95.0)	932.36	99.67
	4	4	2724	89.9 (82.1, 94.6)	72.63	95.87
	5	3	1214	86.0 (69.1, 94.4)	68.178	97.06

Region 1: Center, Region 2: north, Region3: south, Region 4: east, Region 5: west

## Discussion

In this study, the results of original articles were selected by critical appraisal and combined with random effect model. Based on the results, 87% of Iranian population in permanent dentition suffer from at least one type of malocclusions. The high prevalence of malocclusion was also reported in other countries, for example in Pakistan (73.6%), (26) Turkey (96.5%), (27) India (85.5%), (28) Nigeria (88.2%), (29) and Sudan (98.6%), (30) and that are approximately similar to the result of this meta-analysis in Iran.

The prevalence of Class I malocclusion was in the range of range of 40.4%, (31) to 84.3%, (30) in Europe and Africa, respectively. The prevalence of class I malocclusion in Iran was similar to other Asian countries (26–28), and Americans (32). Thirty percent of American children and youths have normal occlusion based on Angle classification and Class I malocclusion (50% to 55%) is by far the largest single group in this population (32), which is close to the result of this study. In comparison with Danish (33), Africans (29, 30, 34), and Latinos (35), Iranian children had fewer Class I malocclusions.

The prevalence of a Class I malocclusion in males (50.35%) in this study was higher than that of females (48.9%). Similar trends were found (33). Contrary to the present result Goose et al (36), and Wood et al (37), showed female predominance in Class I malocclusions.

The prevalence of class II malocclusion was 21% (13.9% division 1 and 4.8% division 2) which was comparable with Caucasians (38). Although this prevalence was higher than in white Americans (39), and was more comparable with western Europeans (33, 36, 40, 41). In addition, Class II malocclusion was the most prevalent in whites of northern European descent (32). The pooled prevalence of Class II in this study was similar to the findings of the studies conducted in Asian countries such as Pakistan (26), Turkey (27), and India (28). We can point to racial, ethnic, cultural and nutritional similarities. Class II malocclusions are less prevalent (5%–10%) in isolated populations such as American Indians (42), Eskimos (37), and native Australians (43). Iranian children also showed a higher prevalence of Class II malocclusions compared with Egyptians (44), Lebanese (45), and blacks and black Africans (29, 30, 46–48).

Class III problem was the least prevalent malocclusion (5.5%) in Iranian populations which some of them were pseudo class III but this was more prevalent than the Caucasians and less than Eastern Asian populations (38, 49). It was comparable with Latinos (35), and black Americans (50). This is probably due to the racial differences. Previous studies in Lebanon (45), and Egypt (44), also yielded a higher prevalence of Class III malocclusions compared with white Americans or Caucasians. Class III problems were reported in Americans less than 1% that represents a very small proportion of the total malocclusions (32). On the other hand, studies in Chinese (15.7%) and Malaysian (16.6%) groups showed a much higher mean prevalence rate than other racial groups (51–55).

According to analysis, minimum and maximum prevalence of malocclusion are in the South and East of Iran, respectively. Although variation of number and sample sizes in the studies, cause to impossibility of a subtle argumentation on geographical distribution.

One of limitations of the study was incomplete report of data in some articles. Reporting of sexual distribution of malocclusions and prevalence of subdivisions of Class II and class III malocclusions are the examples of incomplete data. Another limitation was the lack of enough study about prevalence of malocclusion in central and western regions of Iran.

## Conclusion

The pooled prevalence of malocclusion is about 87% of Iranian population. Class I, II, and III malocclusions were reported 50.7%, 21%, and 5.5%, respectively.

## Ethical considerations

Ethical issues (Including plagiarism, informed consent, misconduct, data fabrication and/or falsification, double publication and/or submission, redundancy, etc.) have been completely observed by the authors.
